# Fully Integrated Microfluidic Platform for Multiplexed Detection of Hunov by a Dynamic Confined‐Space‐Implemented One‐Pot Rpa‐Lamp System

**DOI:** 10.1002/advs.202306612

**Published:** 2023-12-21

**Authors:** Fumin Chen, Chenang Lyu, Zhao Li, Leshan Xiu, Huimin Li, Yi Xie, Runzhen Cao, Qinqin Hu, Kun Yin

**Affiliations:** ^1^ School of Global Health Chinese Center for Tropical Diseases Research Shanghai Jiao Tong University School of Medicine Shanghai 200025 P. R. China; ^2^ Department of Food Science and Technology School of Agriculture and Biology Shanghai Jiao Tong University Shanghai 200240 P. R. China; ^3^ Stake Key Laboratory on Integrated Optoelectronics Institute of Semiconductors Chinese Academy of Sciences Beijing 100083 P. R. China; ^4^ College of Materials Science and Opto‐Electronic Technology University of Chinese Academy of Sciences Beijing 100049 P. R. China

**Keywords:** dynamic confined space, HuNoV diagnosis, integrated microfluidic chip, one‐pot RPA‐LAMP, portable detection platform

## Abstract

Human norovirus (HuNoV) is the leading cause of nonbacterial acute gastroenteritis, which is highly infectious, rapidly evolving, and easily transmitted through feces. The accurate and early detection of HuNoV subtypes is essential for effective treatment, early surveillance, risk assessment, and disease prevention. In this study, a portable multiplex HuNoV detection platform that combines integrated microfluidics and cascade isothermal amplification, using a streamlined protocol for clinical fecal‐based diagnosis is presented. To overcome the problems of carryover contamination and the incompatibility between recombinase polymerase amplification (RPA) and loop‐mediated isothermal amplification (LAMP), a Dynamic confined‐space‐implemented One‐pot RPA‐LAMP colorimetric detection system (DORLA) is developed by creating a hydrogen bond network. The DORLA system exhibits excellent sensitivity, with detection limits of 10 copies µL^−1^ and 1 copy µL^−1^ for HuNoV GI and GII, respectively. In addition, a portable diagnostic platform consisting of a thermostatic control module and an integrated 3D‐printed microfluidic chip for specific HuNoV capture, nucleic acid pretreatment, and DORLA detection, which enables simultaneous diagnosis of HuNoV GI and GII is developed. A DORLA‐based microfluidic platform exhibits satisfactory performance with high sensitivity and portability, and has high potential for the rapid point‐of‐care detection of HuNoV in clinical fecal samples, particularly in resource‐limited settings.

## Graphical abstract

Herein, a dynamic confined space implemented one‐pot recombinase polymerase amplification and Loop‐mediated isothermal amplification colorimetric detection system (DORLA) is established by creating a hydrogen bond network, which can achieve a simultaneous diagnosis of Human norovirus GI and GII on the portable diagnostic platform.

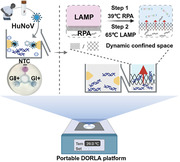

## 1 Introduction

Human norovirus (HuNoV) is a major cause of acute viral gastroenteritis worldwide.^[^ ^1^ ^]^ It is responsible for ≈267 million infections and more than 200 thousand deaths annually. The GI and GII genotypes of HuNoV are widespread and are the main cause of global outbreaks.^[^ ^1^, ^2^ ^]^ The ability to simultaneously detect of HuNoV GI and GII will contribute to the development of effective treatment and prognosis strategies.^[^ ^3^, ^4^ ^]^ Considering the rapid transmission of HuNoV, timely and sensitive identification is critical for the effective prediction and control of HuNoV infections.^[^ ^5^ ^]^ Recent technological advances have facilitated the emergence of laboratory tests for HuNoV detection, such as the reverse transcription‐quantitative polymerase chain reaction (RT‐qPCR),^[^ ^6^ ^]^ which is widely considered to be the gold standard.^[^ ^7^ ^]^ However, RT‐qPCR typically requires various manual procedures and bulky equipment, such as thermocyclers, which pose a major problem for the timely diagnosis required for effective treatment and outbreak containment, especially in resource‐limited settings.^[^ ^8^, ^9^ ^]^

Isothermal amplification assays, such as recombinase polymerase amplification (RPA) and loop‐mediated isothermal amplification (LAMP), have been developed as the alternatives to RT‐qPCR for the detection of HuNoV, and exhibit the distinct advantages of high‐sensitivity, rapidness, and simplicity.^[^ ^5^, ^10^, ^11^, ^12^, ^13^ ^]^ For example, a real‐time RT‐RPA method has been established to detect HuNoV with a detection limit of 1.66 × 10^2^ copies µL^−1^ in only 30 min.^[^ ^11^ ^]^ The LAMP hybridization chain reaction method has demonstrated the capability to successfully detect 30 copies of HuNoV in fecal samples.^[^ ^13^ ^]^ To further improve the detection sensitivity, a two‐step detection strategy that combines RPA and LAMP has been reported; however, it may increase the risk of false positives towing to aerosol contamination.^[^ ^4^, ^14^ ^]^ To address this issue, Song et al. performed RPA and LAMP on the lid and bottom of a tube, respectively. In this case, an additional centrifugation step was required to mix the two reactions.^[^ ^15^ ^]^ The development of a simple one‐pot detection method that can cascade the RPA and LAMP reactions without additional complicated procedures remains a major challenge.

Microfluidics has emerged as a powerful and promising tool for next‐generation disease diagnostics, offering outstanding advantages such as portability, automation, cost‐effectiveness, and point‐of‐care testing.^[^ ^17^ ^]^ In addition, multiple targets can be detected in parallel by utilizing spatially separated detection sites.^[^ ^18^ ^]^ In an earlier study, a simple HuNoV capture method was developed based on the histo‐blood group antigens (HBGAs) in porcine gastric mucin, which can identify HuNoV in various types of samples, including fecal matter, water, and strawberry.^[^ ^19^, ^20^ ^]^ The integration of sample pretreatment into microfluidic devices enables the rapid on‐site detection of HuNoV.^[^ ^21^ ^]^ Therefore, the development of a fully integrated microfluidic detection system that includes selective target concentration, virus lysis, nucleic acid purification, and one‐pot cascading RPA and LAMP reactions is crucial for the sensitive and multiplexed detection of different HuNoV subtypes, particularly from complicated clinical samples.

**Figure 1:**
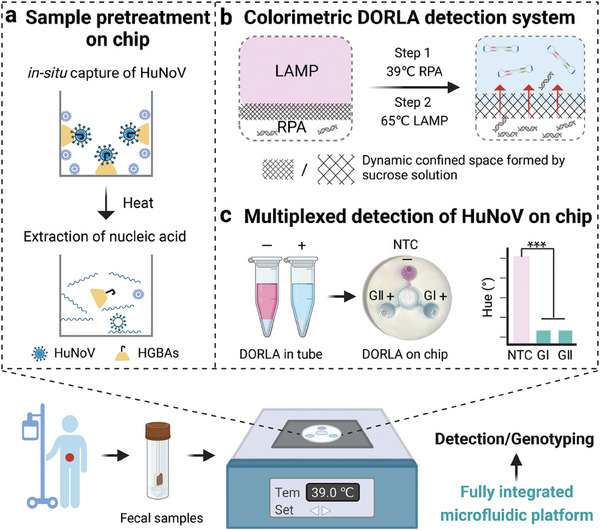
Schematic illustration of the fully integrated microfluidic platform for the multiplexed detection of HuNoV GI and GII in fecal samples based on the dynamic confined space implemented one‐pot RPA‐LAMP system (DORLA). a) Sample pretreatment on‐chip, including in‐situ capture and extraction of nucleic acids of HuNoV. b) Colorimetric DORLA detection system facilitated by sucrose hydrogen bond network (HBN). c) Multiplexed detection of HuNoV on a microfluidic chip.

Here, we report a fully integrated microfluidic detection platform that combines sample pretreatment and isothermal amplification‐based diagnostics with a streamlined workflow for multiplexed detection of HuNoV from clinical fecal samples (**Figure** **1**). To the best of our knowledge, this is the first study to achieve Dynamic confined‐space‐implemented One‐pot RPA‐LAMP colorimetric detection (DORLA) through the construction of a sucrose hydrogen‐bond network (HBN) (Figure 1b). With the assistance of the high‐density and the temperature‐dependent diffusion capacity of HBN, the DORLA system resolved the incompatibility between RPA and LAMP to achieve ultrahigh sensitivity without carryover contaminations.^[^ ^22^, ^23^ ^]^ In addition, we established a portable diagnostic platform composed of a thermostatic control module for DORLA detection and a fully integrated 3D‐printed microfluidic system to realize HuNoV capture, nucleic acid purification, and the simultaneous detection of HuNoV GI and GII (Figure 1a,c). Therefore, the DORLA‐based system is a valuable testing tool for the sensitive and multiplexed detection of HuNoV subtypes and demonstrates high potential for application to point‐of‐care diagnosis in resource‐limited settings in combination with a fully integrated microfluidic detection platform.

## 2 Results and Discussion

### 2.1 Overall Design of the DORLA System

**Figure 2:**
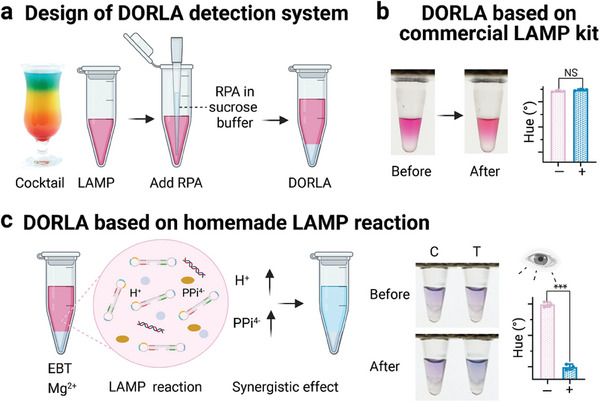
Design of DORLA assay. a) The dynamic confined space reaction inspired by the cocktail. b) The DORLA system by a combination of RPA reaction and the commercial LAMP. c) The DORLA system using homemade LAMP solution. NS means *p* > 0.05, ^***^ means *p* <  0.001 (*n* = 3).

Cascading RPA and LAMP reactions can improve the sensitivity of molecular diagnosis. In addition, multiplexed detection can be realized by pre‐amplification of different targets during the RPA reaction and subsequent LAMP reaction with specific primers.^[^ ^4^ ^]^ However, the poor biocompatibility of the RPA and LAMP reactions, which may induce aerosol contamination from amplicon‐rich tubes during two‐step amplification, still poses a major challenge.^[^ ^16^ ^]^ Inspired by the layer separation in a cocktail, a dynamic diffusion system, DORLA, was used to link the RPA and LAMP reactions in one tube (Figure 1b and **2** **a**). DORLA involves the following attributes: 1) the bottom phase of the DORLA system provides an independent reaction environment for the RPA reaction due to the dynamic confined space formed by HBN^[^ ^24^, ^25^ ^]^; 2) the small nucleic acid amplicons generated in the bottom phase are dynamically diffused into the top phase and trigger the LAMP reaction for colorimetric detection; 3) at the RPA reaction temperature (e.g., 37–42 °C), HBN is dense and can provide a more independent reaction environment for the RPA reaction.^[^ ^22^, ^23^ ^]^ When the temperature is increased to the LAMP reaction temperature (e.g., 65 °C), HBN becomes evacuated, which is more favorable for the RPA amplicons to pass through the HBN to the top phase and thus triggers LAMP detection.^[^ ^26^, ^27^ ^]^

To validate the design of the DORLA system, we first verified the dynamic diffusion between the two phases at the RPA and LAMP reaction temperatures. The sucrose solution containing the dye was added to water (top phase) to form the bottom phase. As shown in Figure S1 (Supporting Information), dye molecules diffused more easily to the top phase at 65 °C than at 39 °C in sucrose solution. The results indicated that the HBN of the sucrose solution offered a relatively isolated environment at low temperatures. In contrast, at high temperatures, HBN became inattentive and merged with the upper phase owing to the increased dynamic diffusion.

Next, we investigated the DORLA method for HuNoV detection using specific LAMP primers and synthetic plasmid templates^[^ ^28^, ^29^ ^]^ (Tables S1 and S2, Supporting Information). First, HuNoV GI and GII were detected using a commercial LAMP kit (WarmStart Colorimetric LAMP 2X Master Mix). As shown in Figure S2 (Supporting Information), 10^4^ copies µL^−1^ HuNoV GI and GII were detected and the solution color changed from red to yellow. Then, we established the DORLA system using a combination of RPA reaction and the commercial LAMP, and successfully obtained a fluorescent signal (Figure S3, Supporting Information). However, no color change occurred after incubation at 39 °C for 15 min and 65 °C for another 30 min (Figure 2b). The colorimetric detection of commercial LAMP kits is based on a pH indicator that can detect the change in pH value caused by the byproducts of H^+^ ions during the LAMP detection. However, in the DORLA system, the RPA buffer may influence a change in the pH value, such that the reaction cannot be detected using the pH indicator. To induce a colorimetric change that can be easily detected by the naked eye, we established a DORLA system using a homemade LAMP solution in combination of RPA reaction.^[^ ^30^ ^]^ The large quantities of byproducts, including PPi^4−^ and H^+^ ions, synergistically influence the color change of indicator (EBT) during amplification, enabling sensitive colorimetric detection (Figure 2c). The color change was captured by the camera and analyzed by the “Hue Analyzer” app for further analysis.

### 2.2 Detection Performance of the DORLA System

The reaction system was optimized to achieve the best DORLA performance. As the efficiency of the DNA polymerase and the color of EBT are affected by Mg^2+^, the optimal Mg^2+^ concentration for the LAMP reaction was investigated for the best LAMP reaction. As shown in Figure S4a (Supporting Information), the optimal concentration of Mg^2+^ was 8 mm. Next, the pH and EBT concentrations were investigated. As shown in Figure S4b (Supporting Information**),** the maximum color change was observed at pH 8.8. The optimal EBT concentration to achieve the maximum ΔHue value was 72 mm (Figure S4c, Supporting Information). We also investigated the effects of the volume ratio of RPA to LAMP from 1:9 to 9:1 on the DORLA detection performance. As shown in Figure S4d (Supporting Information**),** the largest ΔHue occurred when the volume ratio of RPA to LAMP was 3:7, indicating that the RPA reaction can improve the detection capacity at the optimal ratio, although large volumes of RPA solution in the DORLA detection system inhibit accurate detection.

**Figure 3:**
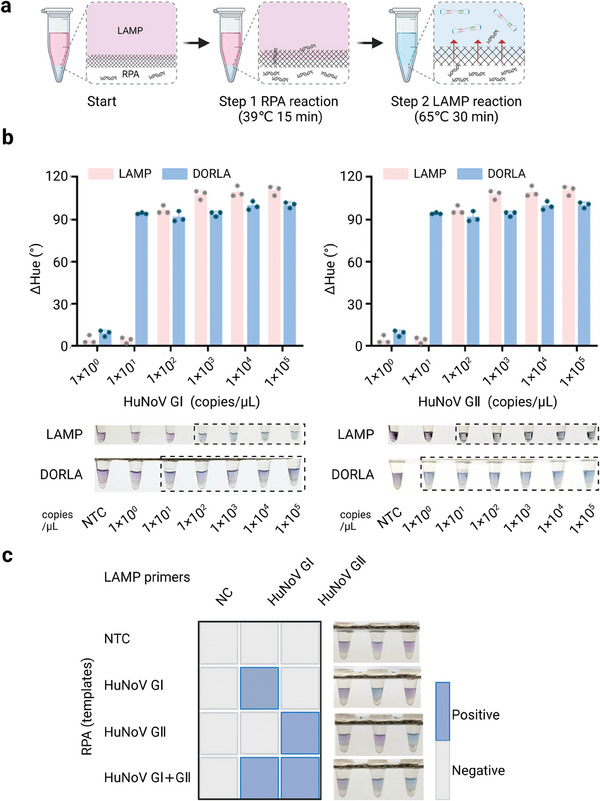
The sensitivity and specificity of the DORLA system for the detection of HuNoV. a) The detection workflow of the DORLA system. b) The sensitivity of the DORLA system. c) The specificity of the DORLA system. Note: NTC‐no template control, NC‐no LAMP primers control.

Next, the sensitivity of the DORLA system was investigated after incubation at 39 °C for 15 min and 65 °C for another 30 min (**Figure** **3**a), and the ΔHue values were used to evaluate the detection performance. As shown in Figure 3b, the limits of detection (LODs) of the DORLA system are 10 copies µL^−1^ and 1 copy µL^−1^ for HuNoV GI and GII, respectively. In comparison to the homemade LAMP reaction without pre‐amplification, in which the LODs were 100 copies µL^−1^ for HuNoV GI and 10 copies µL^−1^ for HuNoV GII, the DORLA system exhibited a ten‐fold improvement in the detection sensitivity, which was attributed to the two‐stage amplification process.^[^ ^19^, ^20^ ^]^ These results indicate that the established DORLA system successfully addresses the poor compatibility between RPA and LAMP reactions without compromising the sensitivity, and reduces the potential carryover contaminations resulting from the two‐step amplifications.

To verify the specificity of the DORLA system, the target HuNoV genotypes was detected in the presence of other HuNoV genotypes. As shown in Figure 3c, a positive signal was generated only in the presence of the target HuNoV genotype and its specific LAMP primers, which indicated the satisfactory specificity of the established DORLA system. In addition, multiple detections were achieved by DORLA with specific HuNoV GI or GII LAMP primers when HuNoV GI and GII were simultaneously present in the solution. This remarkable specificity of the DORLA system is due to the two‐stage cascade amplification that effectively addresses the problem of potential false‐positive results commonly associated with RPA or LAMP and provides the opportunity for the multiplex detection of HuNoV GI and GII.

### 2.3 HuNoV Genotyping Using a Portable DORLA Platform

**Figure 4:**
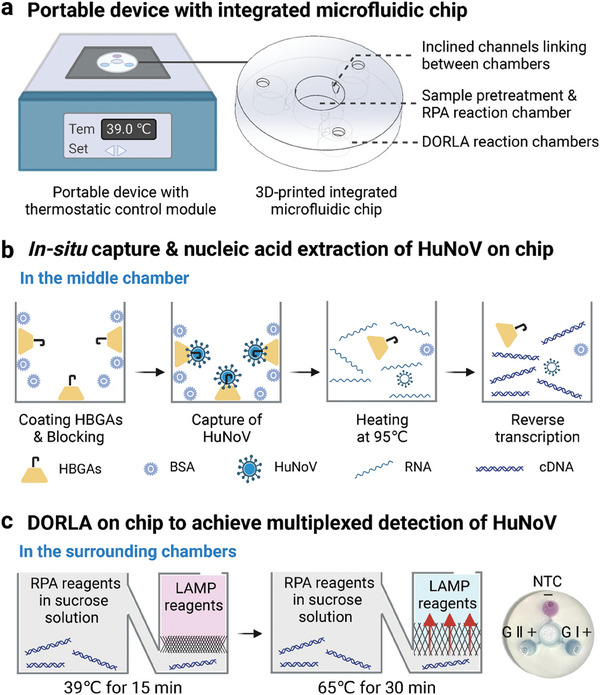
Design of the portable DORLA platform. a) A diagram of a portable DORLA platform with a heater and a fully integrated microfluidic chip. b) The flowchart of in situ capture and nucleic acid extraction of HuNoV on the microfluidic chip. c) Multiplexed detection of HuNoV GI and GII on the portable DORLA platform.

To facilitate the application of DORLA detection in diverse settings, we established a portable DORLA platform that included a heater and a fully integrated microfluidic chip (**Figure** **4**a). The internal structure of the heater, which provided an accurate and stable temperature for DORLA detection, is shown in Figure S5 (Supporting Information). To achieve integrated and multiplexed detection, a microfluidic chip that combined sample pretreatment and a DORLA system was developed. The chip has a diameter of 20 mm and a height of 3.5 mm. It consists of one chamber (*d* = 6 mm) in the center for sample pretreatment and the RPA reaction, and three chambers (*d* = 4 mm) at the edge of the DORLA detection system (Figure 4a; Figure S6, Supporting Information). In addition, three inner inclined channels connecting the middle chamber to the surrounding chambers, ensured integrated sample preparation and DORLA detection on chip. The integrated microfluidic chip was fabricated using a clear resin material owing to its high‐temperature resistance and excellent transparency. To improve the biocompatibility and enhance the colorimetric signals, the chip was coated with 2.5% PVA, which has been validated by previous studies.^[^ ^31^, ^32^ ^]^

To investigate the performance of the portable DORLA platform, the multiplexed detection of HuNoV GI and GII was performed using an integrated microfluidic chip on a homemade heater. Our previous study demonstrated that receptor HBGAs can be used to coat the surface of a qPCR tube to capture HuNoV and realize sensitive receptor‐mediated in situ capture RT‐qPCR (ISC‐RT‐qPCR) detection.^[^ ^33^, ^34^, ^35^ ^]^ To investigate the HuNoV capture ability by coating HBGAs on the resin material, we performed ISC‐RT‐qPCR using PCR tubes modified with resin. As shown in Figure S7 (Supporting Information), there was no significant difference in the cycle thresholds between ISC‐RT‐qPCR and resin‐ISC‐RT‐qPCR, indicating that the HBGAs‐coated resin effectively captured HuNoV. Next, the in situ capture and nucleic acid extraction of HuNoV were performed in the middle chamber of the chip, which included HBGAs coating, HuNoV capture, nucleic acid release, and reverse transcription (Figure 4b). LAMP reagents with specific HuNoV primers were added to the three surrounding chambers for the DORLA colorimetric detection. A high‐density RPA solution (10% sucrose) with both HuNoV GI and GII RPA primers was added to the middle chamber and flowed to the bottom of the LAMP reagents in the surrounding reaction chambers. The microfluidic chip was first incubated at 39 °C for per‐amplification of the target nucleic acids by RPA reaction. When the temperature reached 65 °C, the HBN of the sucrose solution was evacuated, which allowed the RPA amplicons to diffuse easily into the upper phase and trigger colorimetric LAMP detection. As shown in Figure 4c, HuNoV GI and GII were simultaneously detected in the surrounding reaction chambers on the microfluidic chip, indicating that the DORLA detection system was successfully implemented on a portable DORLA platform and achieved multiplexed colorimetric detection. In addition, because the HBGAs can also capture rotavirus particles, we detected rotavirus using the portable DORLA platform to further verify the specificity performance. As shown in Figure S8 (Supporting Information), we did not observe significant colorimetric signals when detecting rotavirus, which can be attributed to the high specific RPA and LAMP primers to HuNoV.

### 2.4 Detection of HuNoV in Clinical Samples

**Figure 5:**
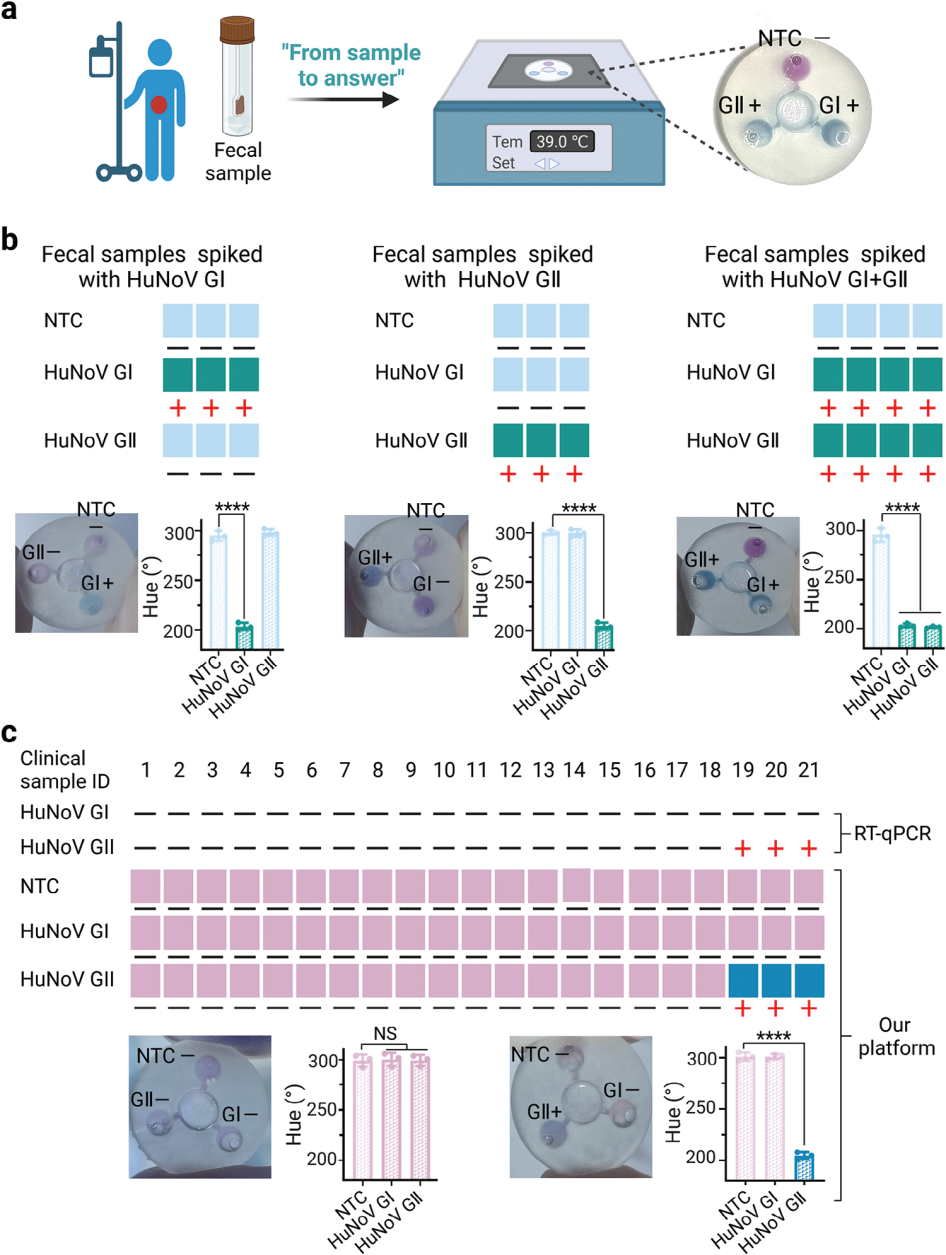
The detection of HuNoV in clinical samples on portable DORLA platform. a) Schematic diagram. b) The performance of the portable DORLA platform on the diagnosis of spiked human fecal samples. c) The detection of HuNoV in real clinical fecal samples using a portable DORLA platform. Note: NTC‐No Template Control; NS means *p* > 0.05, and ^***^ means *p* < 0.0001.

To evaluate the reliability of the portable DORLA platform, HuNoV was detected in clinical samples (**Figure** **5**a). First, we analyzed human fecal samples spiked with 10^5^ copies µL^−1^ HuNoV GI or/and GII templates. As shown in Figure 5b, significant color changes from fuchsia to blue were observed in the chambers with positive results, accompanied by a decrease in ΔHue values. Then, we investigated the performance of the portable DORLA platform for detecting HuNoV in clinical fecal samples collected from Ruijin Hospital North, Shanghai Jiao Tong University School of Medicine. These samples were first added to the middle chamber for the in situ capture of HuNoV, nucleic acid extraction, and detection by DORLA on the chip. As shown in Figure 5c, our platform could be used to detect HuNoV in positive clinical samples, with a color change from fuchsia to blue in the reaction chambers. In contrast, no color changes were observed for the negative samples. The results are in good agreement with those obtained using RT‐qPCR method with spin column‐based RNA extraction kits. We have demonstrated that the portable DORLA platform is suitable for the simple, rapid, and multiplexed detection of HuNoV in complicated bio‐samples with excellent specificity.

As the portable DORLA platform integrates sample pretreatment and colorimetric DORLA detection, it is suitable for performing the on‐site diagnosis of HuNoV without bulky equipment in a centralized laboratory. The total cost of DORLA detection, including disposable microfluidic chips and reaction reagents, is less than two dollars, which is affordable in resource‐limited settings. In addition, the total detection time for DORLA is less than 1 h, indicating that the DORLA system has high potential for application to clinical screening and point‐of‐care diagnosis of HuNoV.

## 3 Conclusion

In this study, we proposed a portable multiplexed HuNoV detection platform consisting of a fully integrated microfluidic chip and a DORLA system. With the dynamic confined‐space‐implemented one‐pot RPA‐LAMP colorimetric detection benefitting from an HBN, the DORLA detection system not only addresses the challenges of poor compatibility and carry‐over contamination of the RPA and LAMP reactions, but also provides ultra‐high sensitivity and specificity for the detection of HuNoV. In addition, the specific HuNoV capture and DORLA detection method can be integrated with the microfluidic chip to successfully achieve the simultaneous diagnosis of HuNoV GI and GII in clinical fecal samples. This portable DORLA platform has been confirmed to have satisfactory performance, including high sensitivity, timeliness, and portability, and is a potential diagnostic tool for on‐site or point‐of‐care screening for pathogens, including but not limited to HuNoV. In the future, we aim to simplify the integrated microfluidic platform by using a battery‐powered heating element, which enables to further eliminate the need for external heating equipment and be suitable for on‐site diagnostics.

## Conflict of Interest

The authors declare no conflict of interest.

## Author Contributions

F.C., C.L., and Z.L. contributed equally to this work. F.C. performed experiments, methodology, and data analysis, and wrote the original draft. **C.L**. acquired resources, and performed data analysis. Z.L. acquired resources and wrote the original draft. L.X., H.L., Y.X., and R.C. acquired resources. Q.H. contributed to conceptualization, and supervision, and reviewed & edited the final manuscript. K.Y. contributed to conceptualization, methodology, and supervision, and reviewed & edited the final manuscript, and acquired funding.

## Supporting information

Supporting Information

## Data Availability

The data that support the findings of this study are available on request from the corresponding author. The data are not publicly available due to privacy or ethical restrictions.
