# Supplementary material for: Fully Integrated Microfluidic Platform for Multiplexed Detection of Hunov by a Dynamic Confined‐Space‐Implemented One‐Pot Rpa‐Lamp System
Source: Adv Sci (Weinh). 2023 Dec 21;11(9):2306612. doi: 10.1002/advs.202306612 (PMC10916549; doi:10.1002/advs.202306612)
Supplement: Supplementary file 1 — Supporting Information [file ADVS-11-2306612-s001.pdf]

ADVANCED SCIENCE
Open Access

Supporting Information

for *Adv. Sci.*, DOI 10.1002/advs.202306612

Fully Integrated Microfluidic Platform for Multiplexed Detection of Hunov by a Dynamic Confined-Space-Implemented One-Pot Rpa-Lamp System

Fumin Chen, Chenang Lyu, Zhao Li, Leshan Xiu, Huimin Li, Yi Xie, Runzhen Cao, Qinqin Hu and Kun Yin**

Supporting Information

Fully integrated microfluidic platform for multiplexed detection of HuNoV by a dynamic confined-space-implemented one-pot RPA-LAMP system

Fumin Chen, *Chenang Lyu*, *Zhao Li*, *Leshan Xiu*, *Huimin Li*, *Yi Xie*, *Runzhen Cao*, *Qinqin Hu*[*], and *Kun Yin*[*]

F. Chen, L. Xiu, H. Li, Y. Xie, R. Cao, Q. Hu, K. Yin

School of Global Health

Chinese Center for Tropical Diseases Research

Shanghai Jiao Tong University School of Medicine

Shanghai 200025, P. R. China

E-mail: qinqinhu@sjtu.edu.cn; kunyin@sjtu.edu.cn

C. Lyu

Department of Food Science and Technology

School of Agriculture and Biology

Shanghai Jiao Tong University

Shanghai 200240, P. R. China

Z. Li

Stake Key Laboratory on Integrated Optoelectronics

Institute of Semiconductors

Chinese Academy of Sciences

Beijing 100083, P. R. China

Z. Li

College of Materials Science and Opto-Electronic Technology

University of Chinese Academy of Sciences

Beijing, 100049, P. R. China

1.

2. Experimental Section

1.1. Materials and reagents

Bst 2.0 DNA polymerase (8000 U/mL), $MgSO_4$, deoxynucleotides (dNTPs), and WarmStart® Colorimetric LAMP 2X Master Mix (M1800S) were purchased from New England BioLabs, Inc. (Ipswich, USA). The TwistAmp Basic RPA Kit was purchased from TwistDx, Inc. (Cambridge, UK). PrimeScript^{TM} RT Reagent Kit (RR047Q, TaKaRa, Japan) and QuantiTect Probe RT-PCR Kit (204443, Qiagen, Valencia, USA) were purchased for the standard RT-qPCR detection. The plasmid templates of HuNoV GI/GII, RPA primers, LAMP primers, and RT-qPCR primers were synthesized by Tsingke Company, Inc. (Beijing, China). The sequence information is shown in Tables S1, S2, and S3 [1, 2]. The tube amplification reactions were carried out in a CFX96 Touch™ Real-Time PCR Detection System (Bio-Rad, Inc., Boston, USA). All the chemicals used in this study such as eriochrome black T (EBT), poly (vinyl alcohol) (PVA), and sucrose were purchased from Sigma-Aldrich, Inc. (St. Louis, USA) or Sinopharm Chemical Reagent Co., Ltd. (Shanghai, China), which were analytical grade or better. Hydrophobic PTFE membrane filters were purchased from Sterlitech, Inc. (Auburn, USA). Clear resin (GPCL02) was purchased from Formlabs, Inc. (Boston, USA).

1.2. Commercial LAMP, homemade LAMP, and DORLA assays

The homemade LAMP reaction contained 0.32 U/μL *Bst* 2.0 DNA polymerase, 5.3 μM primer mix, 1.4 mM dNTPs, 8 mM $MgSO_4$, 72 μM EBT, 1 μL of a plasmid template and 4.8 μL homemade buffer. The homemade buffer was prepared according to reference [3], by mixing 4 μL 1 M KOH, 10 μL 1M $(NH_4)_2SO_4$, 25 μL 2 M KCl, 1 μL Tween 20, and 280 μL ddH_2O. LAMP primer mix was mixed with 5 μL 100 μM F3/B3, 40 μL 100 μM FIP/BIP, and 20 μL 100 μM LF/LB (or LF). Commercial LAMP reactions were performed according to WarmStart® Colorimetric LAMP 2X Master Mix (M1800S) protocols. Both the homemade and commercial LAMP reactions were incubated at 65°C for 45 min and then captured the images by smartphone for analysis.

For the DORLA reaction, 35 μL of LAMP solution was first added into the tube. Then 15 μL of RPA solution with 4 μM F3/B3, 10% sucrose solution, and 2 μL plasmid template was added to the bottom of the tube. The DORLA reaction was first incubated at 39℃ for 15 min and then at 65℃ for 30 min. After the reaction, the colorimetric images were captured by a smartphone for analysis.

1.3. The sensitivity and specificity of the DORLA

To investigate the sensitivity of DORLA, HuNoV GI and GII templates were diluted serially to a final concentration ranging from 1 to 10^5 copies/μL. One microliter of different concentrations of HuNoV GI and GII template was added to the DORLA reaction system, respectively. To investigate the specificity, 10^4 copies/μL of HuNoV GI and GII templates were added into the DORLA reaction system with both HuNoV GI and GII RPA primers and specific LAMP primers. After incubating at 39℃ for 15 min and then at 65℃ for 30 min, the colorimetric images were captured by smartphone for analysis.

2.4. Design of integrated microfluidic chip and thermal-controlled portable device

A cylindrical microfluidic chip (radius = 10 mm, height = 3.5 mm) was designed using SolidWorks software. The chip consisted of three LAMP reaction chambers (r = 2 mm) connected to one sample preparation/RPA reaction chamber in the middle (r = 3 mm). The chip was fabricated with a Form 2 3D printer using the clear methacrylate-based resin and cleaned with isopropyl alcohol (IPA) and ddH_2O under ultrasonic conditions (120 W) for 25 min to remove uncured resin. The chip was then immersed in a 2.5% PVA solution to improve biocompatibility [3, 4].

The portable device with a thermostatic control module was designed to achieve point-of-care (POC) DORLA detection on chip. The device mainly consisted of a heater, a relay, and a temperature controller. A thermoelectric heater (40 mm × 40 mm) was used for heating, which was controlled by the proportional-integral-derivative (PID) temperature controller and relay. The temperature feedback was accomplished via a PT100 thermistor. A powerful miniature fan (12 V × 3 A) with a custom-fabricated aluminum block was placed beneath the thermoelectric cooler to dissipate waste heat.

The isothermal incubation setup can provide a stable reaction temperature.

2.5. Clinical fecal sample preparation

A total of 10 fecal samples were spiked, of which 3 were spiked with 1×10^5 copies/μL of HuNoV GI plasmids, 3 were spiked with 1×10^5 copies/μL of HuNoV GII plasmids, and 4 were spiked with 1×10^5 copies/μL HuNoV GI and GII plasmids. Eighteen negative and three positive clinical human fecal samples were obtained from Ruijin Hospital North, Shanghai Jiao Tong University School of Medicine. All samples were frozen at -80℃ before detection. All clinical samples received approval from the ethics committee (No. 2017-02-01).

2.6. HuNoV *in-situ* capture and nucleic acids extraction on chip

The *in-situ* capture of HuNoV in fecal samples was performed as previously reported [5-7]. Briefly, the sample preparation chamber was coated with 30 μL HuNoV receptor solution (1 mg/mL PGM in 0.05 M carbonate-bicarbonate buffer, pH=9.6) overnight at 4°C, followed by blocking with PBS buffer containing 1% BSA (37°C, 1 h) and washing three times with PBST buffer (0.05% Tween 20 in PBS, pH=7.2). The fecal samples (spiked or clinical) were first diluted to 10-fold volume with PBS buffer and then centrifuged for 5 min at 3000 g. Thirty microliter of the supernatant after the centrifugation was added into the PGM-coated middle chamber and incubated at 37℃ for 1 h. Then the supernatant was thoroughly removed, followed by washing the chamber three times with PBST buffer. The chamber was then filled with 7 μL RNase-free ddH_2O and sealed with polyolefin sealing tape. The sample was heated at 95℃ for 5 min to dissociate virus particles and release the RNA in the chamber and cooled at 4℃. Two microliters of transcribed nucleic acid solution were left in the chamber for the subsequent DORLA detection on the chip.

2.7. Multiplexed DORLA-based detection of HuNoV on chip

The three LAMP reaction chambers were first filled with 10 μL of homemade LAMP solution containing no primer, HuNoV GI, and HuNoV GII LAMP primers, respectively. Thirty-five microliters of RPA solution were added into the middle chamber which had already contained transcribed HuNoV nucleic acids. After sealing

with PCR tape (Microseal® 'B' Film), the integrated chip was placed on the portable device and incubated at 39℃ for 15 min, followed by another 30 min at 65℃ to complete DORLA detection. After the reaction was completed, the colorimetric signal was recorded by a smartphone for further analysis.

2.8. Image analysis and statistics

The Hue values of captured images were extracted by software (e.g., Adobe Photoshop 2021 or our custom Android app dubbed "Hue Analyzer") and analyzed by GraphPad Prism 8 with an unpaired one-way ANOVA test. The Hue values of each image were extracted three times. The results were shown as mean ± standard error. Significance thresholds were set as follows: NS $p > 0.05$, * $p < 0.05$, ** $p < 0.01$, *** $p < 0.001$, and *** $p < 0.0001$.

2.9. RT-qPCR reaction

The RT-qPCR reactions were performed as described in previous studies [5, 6, 8]. In brief, viral RNA was extracted and reverse transcribed using PrimeScript™ RT reagent Kit with gDNA Eraser. The complementary DNA was mixed with 2X one-step RT-PCR buffer III, 8 μM primer mix, probe GI P-1106, probe LZIIP, 5 U/μL TaKaRa Ex Taq HS, and primescript RT enzyme mix II. The amplification protocols were as follows: reverse transcription reaction at 42℃ for 10 min, denaturation at 95℃ for 2 min; qPCR amplification for 45 cycles consisting of denaturation at 95℃ 15 s, annealing at 60℃ for 30 s, and collect fluorescent signal on the real-time PCR machine.

3. Supplementary Tables and Figures

Table S1. The RPA and LAMP primer sequences of HuNoV GI and HuNoV GII

Reaction	Genotype	Primer	Sequence (5'-3')	Ref
RPA	HuNoV GI	F3	CCAGCATGGCAAGCCATGTT	[1]
		B3	CCAACCCAGCCATTATACA	
	HuNoV GII	F3	AGTGGTGGTCTGGAGTTTTA	[2]
		B3	TCAAGTGCCATAACCTCATT	
LAMP	HuNoV GI	F3	CCAGCATGGCAAGCCATGTT	[1]
		B3	CCAACCCAGCCATTATACA	
		FIP	CATTTACAAATTCGGGCAGGCGT TGGATGCGGTTCCATGA	
		BIP	GATGGCGTCTAAGGACGCAGCTG TATTAACCTCCGGCAC	
		LF	AGATTGCGATCTCCTGTCCA	
	HuNoV GII	F3	AGTGGTGGTCTGGAGTTTTA	[2]
		B3	TCAAGTGCCATAACCTCATT	
		FIP	AGATTGCGATCGCCCTCCCATGT TGCCCAGACAAGAGGCCATGTT	
		BIP	TGTGAATGAAGATGGCGTCGAAT TATTGGCCTCTGGTACGAGGTT	
		LF	GTCAGAGAATCTCATCCA	
		LB	ATGACGCCGCTCCATC	

Table S2. The primer sequences used for ISC-RT-qPCR, resin-ISC-RT-qPCR, and RT-qPCR

Genotype	Name	Sequence (5'-3')	Length
	GIF1106	CCATGTTCCGCTGGATGCG	19
HuNoV GI	GIR1106	GCGTCCTTAGACGCCATCATC	21
	GIP-1106	VIC-GCGRTCTCCTGTCCACA-MGB	17
	LZIIF	GTGGGATGGACTTTTACGTGCCAAG	25
HuNoV GII	LZIIR	CGTCAYTCGACGCCATCTTCATTCAC	26
	LZIIP	FAM-AGCCAGATTGCGATCGCC-MGB	18

Table S3 The synthetic plasmids of HuNoV GI and GII

Genotype	Sequence (5'-3')	Position	Length	Ref

HuNoV GI	ATGTGCCAGCATGGCAAGCCAT<br>GTTCCGTTGGATGCGGTTCCATG<br>ACCTTGGTTTGTGGACAGGAGAT<br>CGCAATCTCCTGCCCGAATTTGT<br>AAATGATGATGGCGTCTAAGGA<br>CGCCCCTCAAAGCGCTGATGGC<br>GCAAGCGGCGCAGGTCAACTGG<br>TGCCGGAGGTTAATACAGCTGA<br>CCCCTTACCCATGGAACCTGTGG<br>CTGGGCCAACAACAGCCGTAGC<br>CACTGCTGGGCAAGTTAATATGA<br>TTGATCCCTGGATTGTTAATAAT<br>TTTGTCCAGTCACCTCAAGGTGA<br>GTTCACAATCTCTCCTAACAATA<br>CCCCCGGTGATATTTTGTTTGAT<br>TTACAATTAGGTCCACATCTAAA<br>CCCTTTCTTGTCACATTTGTCCCA<br>AATGTATAATGGCTGGGTTGGG<br>AACA	5262-5673	412 bp	[1]
HuNoV GII	TTAAGAGTGGTGGTCTGGAGTTT<br>TATGTGCCCAGACAAGAGGCCA<br>TGTTTAGGTGGATGAGATTCTCT<br>GACCTCAGCACATGGGAGGGCG<br>ATCGCAATCTTGCTCCCGAGAGT<br>GTGAATGAAGATGGCGTCGAAT<br>GACGCCGCTCCATCAAATGATG<br>GTGCTGCCAACCTCGTACCAGAG<br>GCCAATAATGAGGTTATGGCACT<br>TGAACCGG	4962-5172	211 bp	[2]

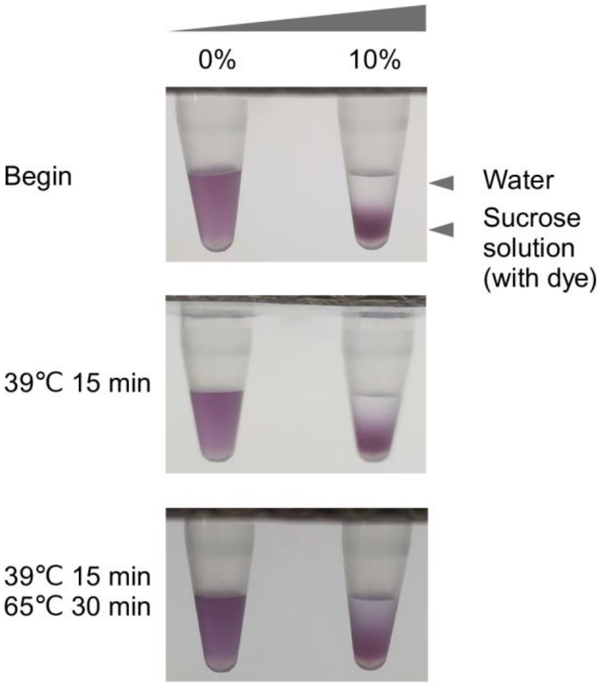

Figure S1. The dynamic diffusion of sucrose solution under different temperatures.

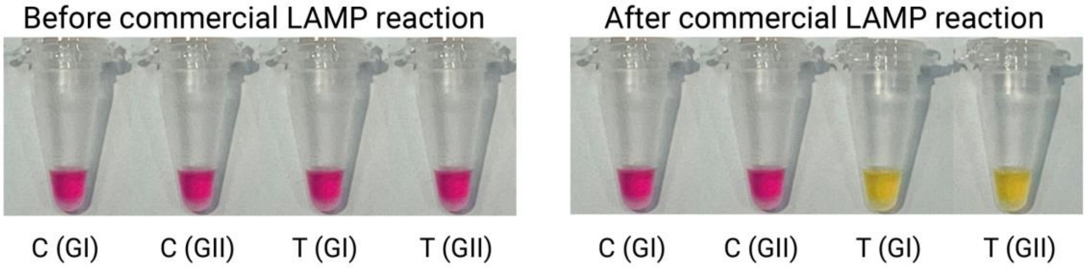

Figure S2. Detection of HuNoV GI and GII using commercial LAMP kit (NEB). After incubation at 65°C for 30 min, the color of test groups with HuNoV GI or GII changed from red to yellow, while the color of control groups stayed red.

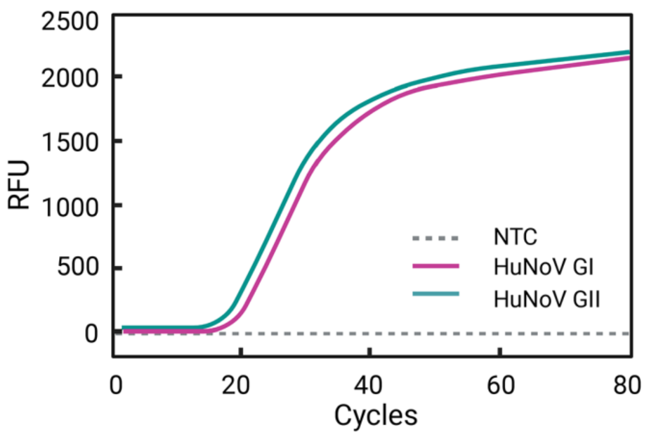

Figure S3. The fluorescent signals of the DORLA system by a combination of RPA reaction and the commercial LAMP for detecting HuNoV GI and GII.

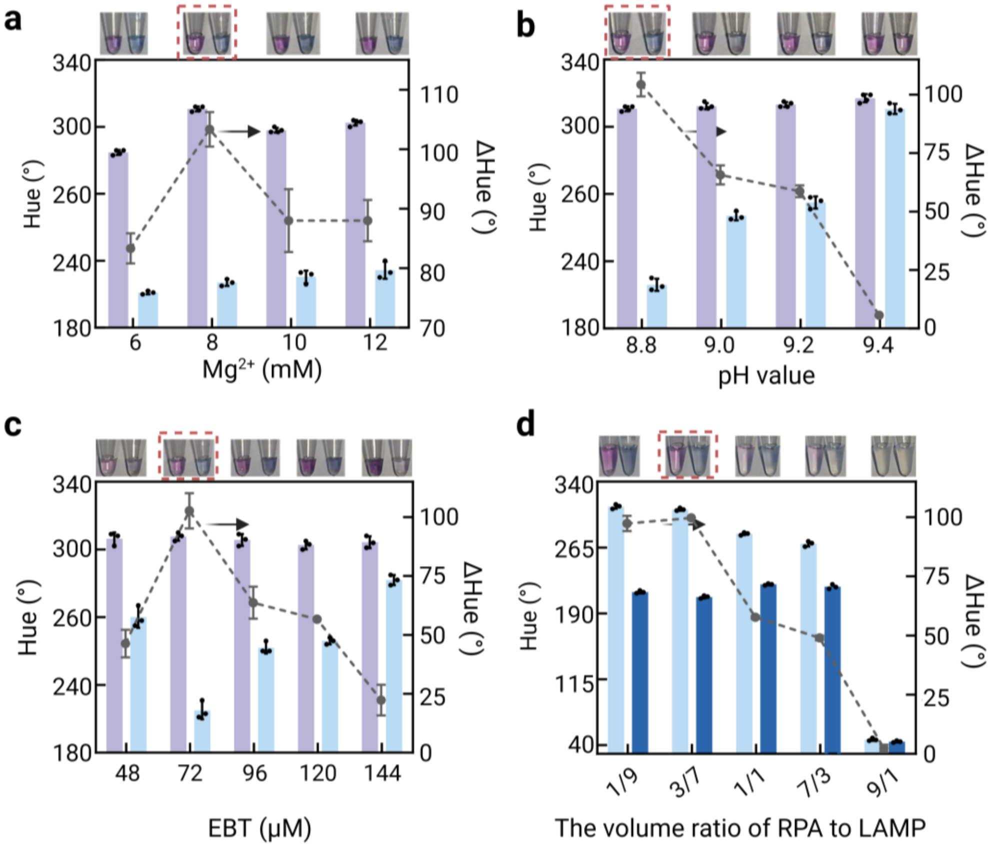

Figure S4. Optimization of the homemade LAMP reaction and the DORLA system. **a)** the optimization of Mg^{2+}. **b)** the optimization of pH value. **c)** the optimization of EBT. **d)** The optimization of the volume ratio of RPA to LAMP.

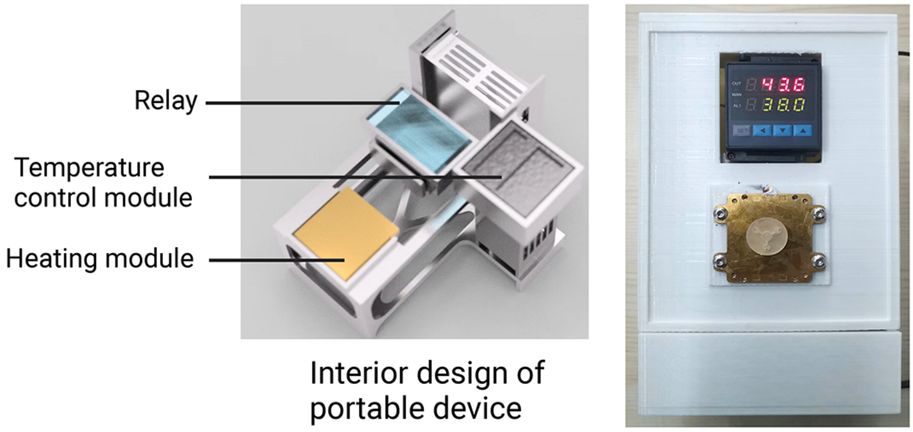

Figure S5 The interior design and physical diagram of the portable device, which contains the relay module, the temperature control module, and the metal heating module.

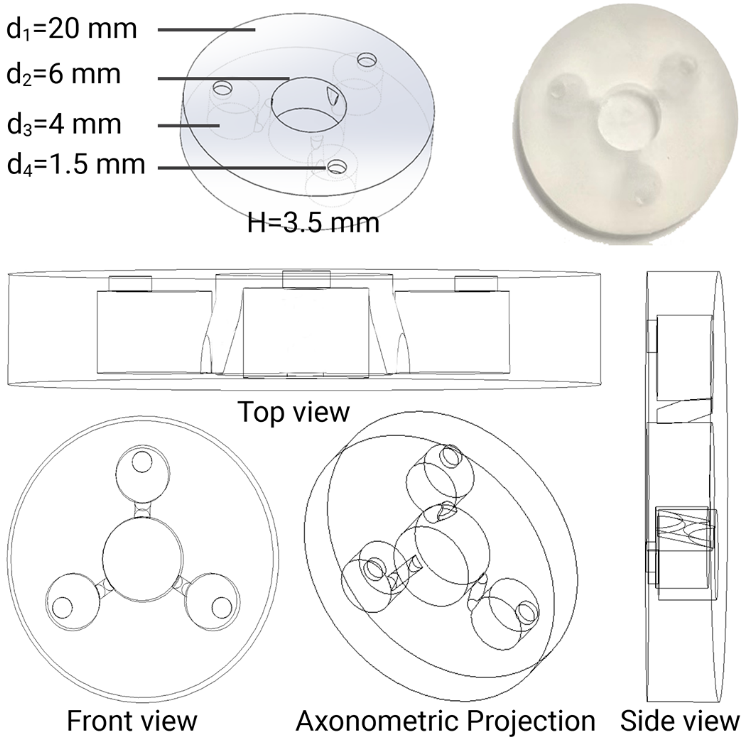

Figure S6 The design of the 3D-printed microfluidic chip (top view, front view, side view, and axonometric projection). The chip is designed with a diameter of 20 mm and a height of 3.5 mm. It contains one cylinder chamber in the middle with a diameter of 6 mm for HuNoV capture, nucleic acid extraction, and RPA reaction. Three cylindrical

chambers with a diameter of 4 mm are located at the edge of the chip, which is used for the DORLA system for simultaneous detection of HuNoV genotypes.

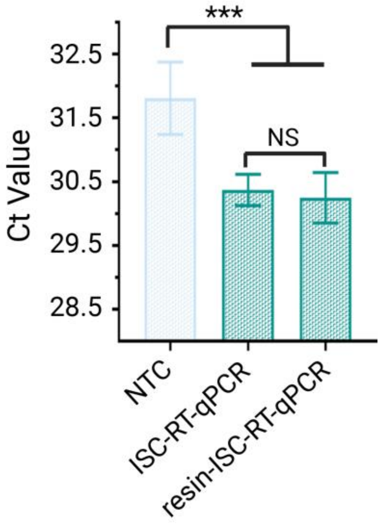

Figure S7. The verification of HBGAs-coated resin can be used to capture HuNoV. PCR tubes and resin-coated PCR tubes were used for ISC-RT-qPCR detection. Note: NS means $p > 0.05$, *** means $p < 0.001$.

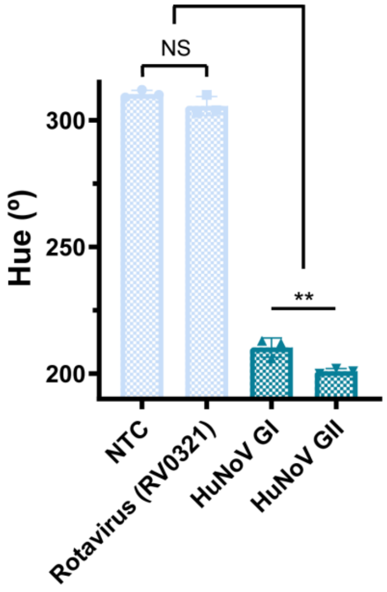

Figure S8. The specific performance in detecting rotavirus using the portable DORLA platform. Note: NS means $p > 0.05$, ** means $p < 0.01$.

References

[1] Fukuda, S., S. Takao, M. Kuwayama, Y. Shimazu, and K. Miyazaki, Rapid detection of norovirus from fecal specimens by real-time reverse transcription-loop-mediated isothermal amplification assay. *J Clin Microbiol*, **2006**. 44(4): 1376-1381.

[2] Yoda, T., Y. Suzuki, K. Yamazaki, N. Sakon, M. Kanki, I. Aoyama, and T. Tsukamoto, Evaluation and application of reverse transcription loop-mediated isothermal amplification for detection of noroviruses. *J Med Virol*, **2007**. 79(3): 326-334.

[3] Yin, K., X. Ding, Z. Xu, Z. Li, X. Wang, H. Zhao, C. Otis, B. Li, and C. Liu, Multiplexed colorimetric detection of SARS-CoV-2 and other pathogens in wastewater on a 3D printed integrated microfluidic chip. *Sens Actuators B Chem*, **2021**. 344: 130242.

[4] Kadimisetty, K., J. Song, A.M. Doto, Y. Hwang, J. Peng, M.G. Mauk, F.D. Bushman, R. Gross, J.N. Jarvis, and C. Liu, Fully 3D printed integrated reactor array for point-of-care molecular diagnostics. *Biosens Bioelectron*, **2018**. 109: 156-163.

[5] Wang, D. and P. Tian, Inactivation conditions for human norovirus measured by an in situ capture-qRT-PCR method. *Int J Food Microbiol*, **2014**. 172: 76-82.

[6] Lyu, C., F. Lu, Z. Shi, and D. Wang, Detection of group A rotavirus in oyster tissues by in situ capture RT-qPCR. *Food Control*, **2021**. 127.

[7] Tian, P., D. Yang, L. Shan, Q. Li, D. Liu, and D. Wang, Estimation of Human Norovirus Infectivity from Environmental Water Samples by In Situ Capture RT-qPCR Method. *Food Environ Virol*, **2018**. 10(1): 29-38.

[8] Liu, D., Z. Zhang, Q. Wu, P. Tian, H. Geng, T. Xu, and D. Wang, Redesigned

Duplex RT-qPCR for the Detection of GI and GII Human Noroviruses. *Engineering*, **2020**. 6(4): 442-448.
